# Sacral neuromodulation: time to seize the opportunity to collaborate on a ‘de-prioritised’ service?

**DOI:** 10.1007/s10151-023-02785-3

**Published:** 2023-04-01

**Authors:** A. O’Connor, D. Mullins, A. Sharma, G. Faulkner, K. Telford

**Affiliations:** 1 Department of Colorectal Surgery, Wythenshawe Hospital, Manchester University NHS Foundation Trust, 2nd Floor Acute Block, Southmoor Road, Manchester, M23 9LT UK; 2 Faculty of Biology, Medicine, and Health, The University of Manchester, Manchester, UK

Dear Sir,

Sacral neuromodulation (SNM) transforms the lives of many patients afflicted with faecal incontinence [1] or urinary dysfunction [2] and yet access to this essential treatment has been, and continues to be, severely impacted following the COVID-19 pandemic [3]. Patients with these conditions already experience high rates of anxiety and depression which is only compounded by delays in treatment that can stretch to years rather than months. The recent pelvic floor report recommends ‘seizing the opportunity’ to improve patient care whilst highlighting the many challenges the service faces as patients with benign pelvic floor conditions are ‘de-prioritised’ whilst the health service is overloaded [3]. The report makes recommendations to address these difficulties including deepening collaboration between pelvic floor units, expanding training opportunities, embracing new technologies, and transitioning procedures away from general anaesthesia (GA) in operating theatres to local anaesthesia (LA) in procedure suites [3].

Recognising these challenges, a meeting of 11 high-volume units implanting SNM devices in the North of England was held in November 2022 to discuss the shared experiences of delivering an SNM service following the pandemic. Prior to the meeting, an online survey of practice with responses from eight units revealed many of the challenges outlined in the pelvic floor report published a year earlier. Patients requiring SNM experienced significant delays with limited access to theatre lists and reduced clinic capacity cited as the predominant reasons. Indeed, only two units (25.0%) described their service as having ‘fully recovered’ from the impact of the pandemic. SNM procedures were performed in a mixture of locations with four (50.0%) performing percutaneous nerve evaluation (PNE) or battery exchanges exclusively in operating theatres whilst the remainder performed these in day-case suites or procedure rooms. The mode of anaesthesia varied with the majority (7, 87.5%) performing PNE under LA, but only 4 (50.0%) and 3 (37.5%) units routinely performing battery exchange or SNM implants under LA respectively. Most units (6, 75.0%) employed an SNM specialist nurse to counsel patients for PNE and SNM implantation, but only one of these nurses routinely performed SNM procedures. Further, only one unit (12.5%) described non-consultant grade doctors being involved in the counselling of patients for SNM or being routinely involved in SNM procedures. This is despite five units (62.5%) highlighting staffing as a key challenge for the delivery of their SNM service. And whilst three units (37.5%) described a transition from face to face to telephone appointments, none had embraced video calls or other novel forms of patient communication in the recovery from the pandemic. This variation across the units also included the membership and role of the multidisciplinary teams, and the patient-reported outcome tools used to measure the success of treatment.

Given the nature of specialist SNM services, they can often work in isolation. Perhaps, faced with the scale of the challenges, ‘seizing the opportunity’ should take the form of collaborating to enact lasting change to improve the delivery of SNM care? Even within a group of geographically connected units, there were stark differences in the service they deliver. This likely reflects the experiences of other units across the country given the parallels drawn between our survey and the pelvic floor report. Quality improvement (QI) collaboratives have been used in surgical care before and involve professionals coming together to learn from, and motivate each other to improve the quality of their service [4]. The Royal College of Surgeons’ cholecystectomy quality improvement collaborative (Chole-QuIC) has demonstrated how, with sufficient willingness from participating hospitals and support from stakeholders, the quality of emergency gallstone care can be dramatically improved with decreased times from diagnosis to surgery [5]. Taking inspiration from this QI initiative in the emergency setting where significant challenges exist following the COVID-19 pandemic, such a QI collaborative could help address the difficulties faced in the provision of SNM services for pelvic floor conditions. Clinically led teams sharing and collaborating through an organised QI collaborative on new ways of working, novel technologies, training opportunities, and SNM surgical techniques could help redress the inequitable access many patients face. By engaging with stakeholders outside of the immediate treatment team including anaesthetists, theatre staff, medical technology companies, and hospital management a QI initiative is likely to result in lasting and meaningful change.

Now, more than ever, is the time to address the challenges highlighted in the pelvic floor report and echoed in our shared experiences. We must seize the opportunity to collaborate to effect change for the benefit of patients who frequently find themselves isolated at the back of a long queue to access health services.
